# An Automated Deep Learning Approach for Spine Segmentation and Vertebrae Recognition Using Computed Tomography Images

**DOI:** 10.3390/diagnostics13162658

**Published:** 2023-08-12

**Authors:** Muhammad Usman Saeed, Nikolaos Dikaios, Aqsa Dastgir, Ghulam Ali, Muhammad Hamid, Fahima Hajjej

**Affiliations:** 1 Department of Computer Science, University of Okara, Okara 56310, Pakistan; aqsi96@gmail.com (A.D.); ghulamali@uo.edu.pk (G.A.); 2 Mathematics Research Centre, Academy of Athens, 10679 Athens, Greece; 3 Department of Computer Science, Government College Women University, Sialkot 51310, Pakistan; mhamid@gcwus.edu.pk; 4 Department of Information Systems, College of Computer and Information Science, Princess Nourah bint Abdulrahman University, Riyadh 11671, Saudi Arabia; fshajjej@pnu.edu.sa

**Keywords:** semantic segmentation, medical image analysis, spine, vertebrae recognition

## Abstract

Spine image analysis is based on the accurate segmentation and vertebrae recognition of the spine. Several deep learning models have been proposed for spine segmentation and vertebrae recognition, but they are very computationally demanding. In this research, a novel deep learning model is introduced for spine segmentation and vertebrae recognition using CT images. The proposed model works in two steps: (1) A cascaded hierarchical atrous spatial pyramid pooling residual attention U-Net (CHASPPRAU-Net), which is a modified version of U-Net, is used for the segmentation of the spine. Cascaded spatial pyramid pooling layers, along with residual blocks, are used for feature extraction, while the attention module is used for focusing on regions of interest. (2) A 3D mobile residual U-Net (MRU-Net) is used for vertebrae recognition. MobileNetv2 includes residual and attention modules to accurately extract features from the axial, sagittal, and coronal views of 3D spine images. The features from these three views are concatenated to form a 3D feature map. After that, a 3D deep learning model is used for vertebrae recognition. The VerSe 20 and VerSe 19 datasets were used to validate the proposed model. The model achieved more accurate results in spine segmentation and vertebrae recognition than the state-of-the-art methods.

## 1. Introduction

The treatment and diagnosis of pathological diseases require adequate spine segmentation and vertebrae recognition, which help to plan better spine surgeries [1]. Vertebral segmentation and recognition are key components in the development of computer-aided diagnosis (CAD) applications for the diagnosis and treatment of spine-related diseases. A computer-based system extracts important features from medical images and creates a 3D model of the patient. The surgeon only needs to make minor adjustments to this model to obtain views of the volume from any angle at any depth. As a result, the surgeon may analyze the situation more thoroughly and provide a more precise diagnosis [2]. The accurate identification of vertebral boundaries and the recognition of individual vertebrae from medical images can improve efficiency and accuracy.

Spine segmentation refers to the process of identifying the vertebral boundaries from medical images such as computer tomography (CT), magnetic resonance imaging (MRI), or X-ray imaging. The segmentation of the spine is challenging due to the complex anatomical structure, the variability in the shape and size of the vertebrae, and the presence of surrounding tissues. Different approaches have been proposed in the literature to address these challenges. Some of the commonly used segmentation techniques are atlas-based segmentation [3], region-based segmentation [4], and active contour-based segmentation [5]. A spine segmentation procedure that creates anatomically correct 3D models can be hindered by certain factors such as the anatomic complexity of the spine, image noise, low intensity, and the partial volume effect [6]. Vertebrae recognition refers to the process of identifying and labeling individual vertebrae from segmented spine images. The accurate recognition of vertebrae is important for various clinical applications such as vertebral fracture detection, scoliosis diagnosis, and surgical planning. Different approaches have been proposed in the literature for vertebrae recognition. Some of the commonly used techniques are template matching [7], shape-based recognition [8], and machine-learning-based recognition [9].

The exact vertebral boundaries, however, cannot be easily defined due to the articulation of the vertebrae with one another, which can cause vertebral overlaps in segmentation. Despite growing interest in spine segmentation and vertebrae recognition, reliable and precise spine segmentation approaches are still absent. Furthermore, numerous techniques to appropriately segment spine patients with osteoporosis fractures fail because such patients frequently experience vertebral fractures in various phases and present spinal anomalies. Because the variability of a distinctive shape differs from the mean shape, model-dependent segmentation may not succeed with such fractures.

In this research, a novel deep learning model is introduced for automated and efficient spine segmentation and vertebrae recognition using CT images. This approach works in two phases. First, a cascaded hierarchical atrous spatial pyramid pooling residual attention U-Net (CHASPPRAU-Net) is used for the segmentation of the spine. The model contains residual and cascaded hierarchical atrous spatial pyramid pooling in the encoder part for feature extraction. The CHASPP module focuses on objects in the dataset, while the residual blocks extract deep features from the dataset. Attention modules are used in the decoder part, which helps improve the performance by focusing on regions of interest. The skip connections pass the information from residual blocks to decoder layers. After that, a novel 3D mobile residual U-Net (MRU-Net) deep learning model is used for vertebrae recognition. This model uses a modified version of MobileNetv2, with residual blocks and depthwise convolutions, as a decoder. Three modified MobileNetv2 architectures are used for feature extraction from axial, sagittal, and coronal views of 3D CT images. The features are combined to form a 3D feature map, which is given to the decoder part for final vertebrae recognition. The decoder part of the model is the expansion part of the standard 3D U-Net for final vertebrae recognition. The proposed models achieve better results in spine segmentation and vertebrae recognition compared to other models.

The main contributions of the proposed approach are given below:

1. A novel automated deep learning approach for spine segmentation with residual blocks, an attention module, a U-Net, and cascaded hierarchical atrous spatial pyramid pooling known as CHASPPRAU-Net, which uses CT images.
2. Efficient and robust automated spine vertebrae recognition using MobileNetv2, residual blocks, and a 3D U-Net architecture known as 3D MRU-Net, which uses CT images.
3. The proposed CHASPPRAU-Net model provides efficient and accurate spine segmentation compared to the state-of-the-art methods.
4. The 3D MRU-Net has an optimal number of trainable parameters that have lower computational costs compared to the other 3D deep learning models.

## 2. Related Work

Medical image analysis has been revolutionized through the innovation of deep learning models. Many machine learning models are available for the diagnosis and treatment of diseases, such as brain tumor segmentation [10], diabetic retinopathy detection [11], glaucoma detection [12], and COVID-19 detection [13]. Kim et al. [14] used deep-learning-based approaches, particularly the U-Net architecture [15], to create an application for segmenting the spine using CT images. The data processing tool was built in Python using the Keras library, while the web accessibility was built using the Flask server framework. The U-Net was trained on 310 CT images, validated on 20 images, and tested on 14 images. The model was able to obtain a dice score of 90.40% for binary segmentation.

An automated deep learning approach for spine segmentation was proposed by Zhang et al. [16] by improving the U-Net model’s architecture. MRI images were used for the segmentation of the spine. The proposed improved U-Net architecture reduced the image processing time of the MRI images compared to the FCN and the standard architecture of the U-Net model. The results of this approach were better than those of previous models; however, the test datasets were very small, and the models were tested on a single source. Christian et al. [17] proposed a four-step approach for automated spine segmentation. First, a basic U-Net model was used to segment the spines in MRI images. After that, a multi-class U-Net model was used to generate a fine segmentation, including labels for vertebrae and vertebral body landmarks. After that, a model-based segmentation was initialized to detect the vertebrae. The proposed model achieved better results using a dataset of 147 images.

A pixel-based model using deep learning was proposed for spine segmentation by Kiran et al. [18]. The proposed model was different from the conventional spine segmentation methods and achieved better results in terms of accuracy and precision. A multitask learning model was proposed by Tran et al. [19] for automated spine segmentation and spinal parameter inspection. This approach consisted of two parts: the first part was used for spine segmentation, while the second part was responsible for spine inspection. The model was evaluated using a dataset that was collected and annotated by radiologists. The results of the model were better for spine segmentation as well as spine inspection. Patch-based deep belief networks were created by Qadri et al. to automatically segment vertebrae in CT images [20]. DBNs are deep learning models made up of layered restricted Boltzmann machines (RBMs) [21]. The proposed methodology helps to quantify the differences between classes by automatically selecting features from image patches. Weight initialization is performed using unsupervised learning, whereas weight updates are conducted via supervised fine-tuning.

Binhui et al. [22] proposed a SePiCo (semantic guided pixel contrast) for semantic segmentation using a one-stage adaption network that used learning of class discriminative and class-balanced pixel representations with the increase in self-training approaches. First, the discriminative features were used for centroid-aware pixel contrast. After that, distribution-aware pixel contrast was also used to determine the correct distribution of each semantic category of a labeled dataset. Ban et al. [23] proposed a feature-based algorithm for the medical image registration of 2D and 3D images. Statistical features were extracted using a weighted spatial histogram of gradient directions. The proposed approach was tested on CT images and X-ray images, which improved the accuracy and efficiency of the model. A cascade residual dense network was developed by Duanet al. [24] for the construction of high-quality diffusion-weighted (DW) images using k-space data. The model achieved better results for lung disease using the DW-MRI dataset.

Diniz et al. [25] proposed an approach that was based on template matching and a CNN model with residual blocks. The model was evaluated using a CT database of 36 patients. The model achieved better accuracy results for spine segmentation. Chang et al. [26] proposed a deep learning approach for spine segmentation where the parameters were updated adaptively based on the input image. In seventeen 3D vertebral CT images of the lumbar and thoracic spine, the normal and pathological instances of both systems were calculated in terms of DSC. The results were compared to four distinct models. The robustness of the APCNN and MLPNN was also tested by introducing noise to the images. With a dice score of 95%, the APCNN outperformed the other approaches.

Having reviewed key works on the use of deep learning for segmentation and classification, it needs to be noted that there are still challenges that need to be improved. The biggest problem is the computational costs of the deep learning models, which require more hardware resources to process the training datasets. The testing images of most of the deep learning models are limited. The testing should contain at least 20% of the total dataset so that the performances of the models can be evaluated correctly.

## 3. Materials and Methods

Deep learning is becoming more popular in medical imaging for the diagnosis [27] and treatment of many diseases, mainly by performing segmentation and classification tasks [28]. For image-related problems, convolutional neural networks are widely used for segmentation and classification, and they usually involve a fully convolutional neural network [29] and some encoder–decoder-based architectures such as V-Net [30] and 3D U-Net [31]. There are many applications of semantic segmentation in medical imaging, such as glomeruli segmentation [32,33], autosomal dominant polycystic segmentation using magnetic resonance images (MRI) [34], brain tumor segmentation [10], and vessel segmentation [35].

### 3.1. MobileNetv2

MobileNetv2 [36] is a neural network architecture that contains 53 deep layers. The features are filtered using a lightweight depthwise convolution that extracts fewer trainable parameters. It is specially designed for devices with low computational resources such as mobile phones. This type of model can reduce the computational costs of machine learning models while maintaining accuracy. The complete architecture of MobileNetv2 is shown in Table 1.

It is based on a residual structure in which there are residual connections between the bottleneck layers. The features are filtered using lightweight depthwise convolutions by intermediate expansion layers. The complete architecture of MobileNetv2 contains fully convolutional layers with 32 filters and 19 residual bottleneck layers. MobileNetv2 is used for feature extraction in spine vertebrae recognition, which decreases the number of trainable parameters and subsequently decreases the computational cost.

### 3.2. Structure of Residual Blocks

**Figure 1:**
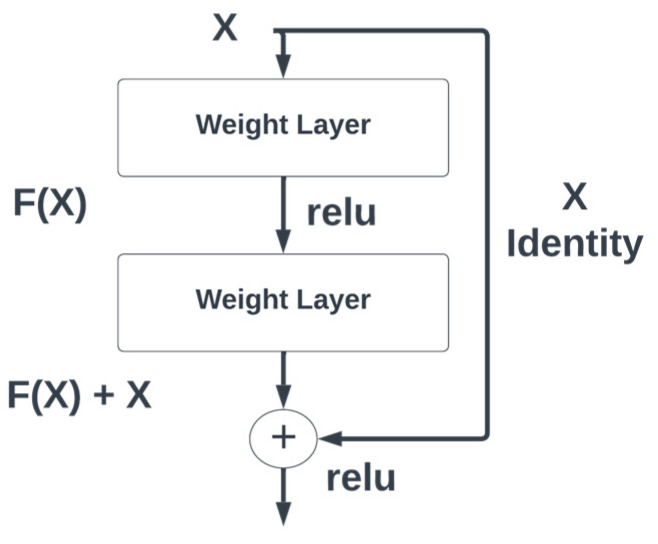
Residual block architecture with skip connections.

Residual blocks [37] are very popular for learning deep features because they make another path to reach other layers of the neural network by skipping some layers. This acts like a stack, where the output of a layer is added to another layer of the network. The complete architecture of the residual blocks is shown in Figure 1.

Residual blocks are used in the encoder part of the proposed model for spine segmentation. The features from residual blocks are passed to the decoder layers using skip connections.

### 3.3. Attention Module

The attention module [38] is widely used in various deep learning tasks, such as image processing, natural language, and video processing, because it has an in-depth understanding of the input. There are two types of attention modules, namely soft attention and hard attention. In soft attention, all data pay attention and calculate the corresponding weight without setting conditions, whereas hard attention filters the unqualified part of the data after the calculations. The general attention module performs the following operations:

Each query vector (*q* = *S_t_* − 1) is matched with database keys to calculate the value of the score. It is considered a dot product of a query by matching with each key vector (*k_i_*).

*e_q,ki_ = q.k_i_* (1)

The weighted sum of vectors (*V_ki_*), where every value is joined with a corresponding key, is computed to obtain the generalized attention:

*Attention* (*q*, *K*, *V*) = ∑*_i_α_q,ki_V_ki_* (2)

**Figure 2:**
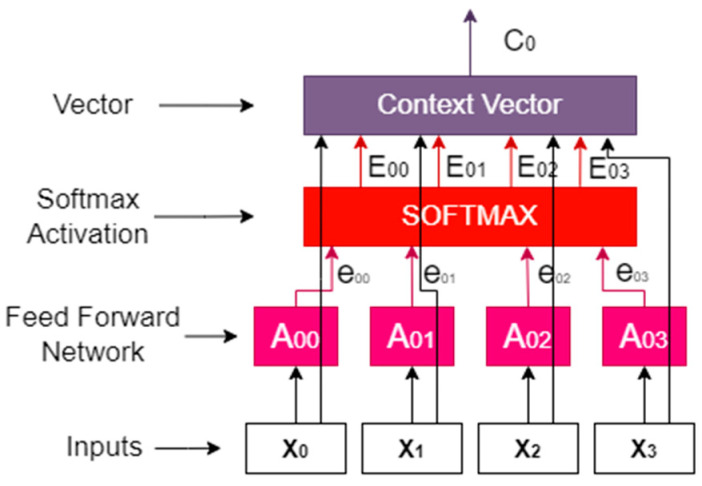
Attention module architecture used in this research.

Each input word is attributed its own key, value, and query. Such vectors are generated by multiplying specific words with three different weight matrices that are generated during the training process. Basically, when the generalized attention mechanism is provided with a string of words, it evaluates each key in the database using the query vector assigned to a particular word in the string. This depicts the relationship between the word under examination and the other words in the sequence. The values are then scaled in accordance with the attention weights to maintain the focus on the query-relevant terms. As a result, the term under consideration receives an output of attention. A general mechanism of an attention module is shown in Figure 2.

To focus on the region of interest, the attention module is used in the decoder part of the proposed model for spine segmentation. The attention modules improve the accuracy of the model.

### 3.4. Atrous Spatial Pyramid Pooling Modules (ASPP)

**Figure 3:**
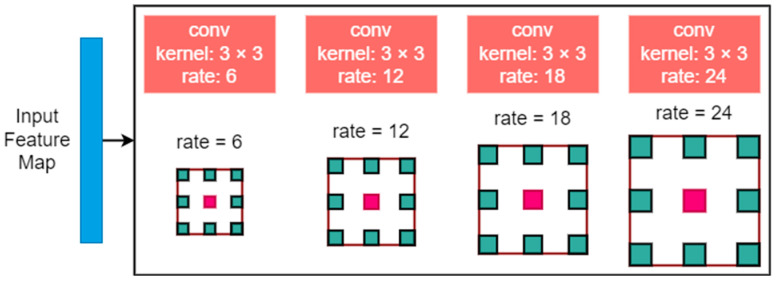
Atrous spatial pyramid pooling module used in this research.

ASPP [39] is a module used for semantic segmentation in which a feature layer can be resampled with different convolution rates. This helps to obtain useful image information at multiple scales and preserve more important information. Besides resampling features, it is implemented using multiple atrous convolutional layers with different sampling rates. The complete working of the ASPP module is shown in Figure 3.

With the help of different convolution rates, the local information from the feature map can be used to improve the performance of the algorithm.

Limitations: As ASPP provides better results using different convolution rates and extracts features at multiple resolutions, it has some limitations [40]. The sampling scope is limited due to the fact that the ASPP is not applicable in some conditions: (1) The target object is in a large distribution or is very disconnected. For objects in a limited scope, ASPP performs well and extracts key information. If the components of an object are split, the ASPP cannot extract global information from them. (2) The contextual information that can provide auxiliary information, which helps to discriminate local patches, is arbitrarily scattered in the image.

### 3.5. Cascaded Hierarchical Atrous Spatial Pyramid Pooling Module

**Figure 4:**
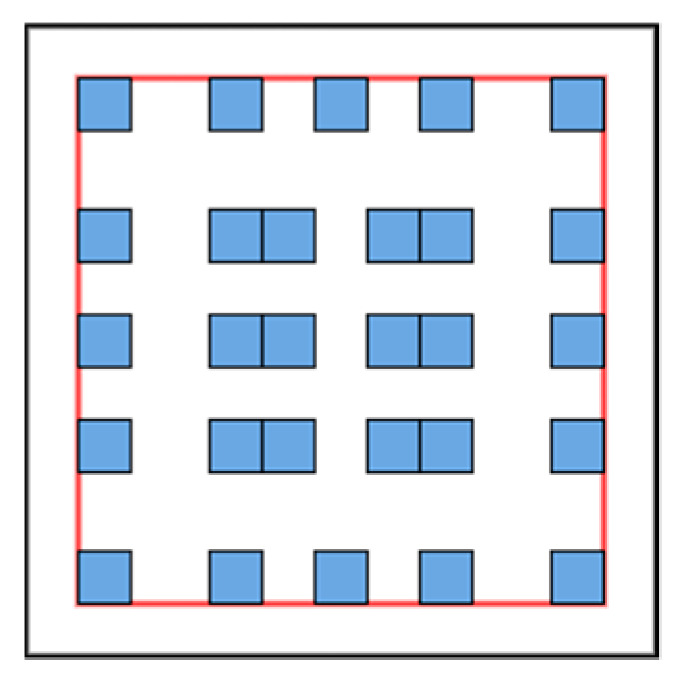
Sampling points of CHASPP. Red donates the receptive field, and blue denotes sampling points.

To solve the problem of the ASPP, a new cascaded hierarchical ASPP was proposed [40] that increases the number of sampling points inside the receptive field. The structure of the ASPP was modified into a two-level hierarchical structure using a one-root atrous convolution and three-branch convolution layers with small convolution layers. The unique features of the sampling area can be extracted easily and can be used in the training process. The density of the sampling points of CHASPP is shown in Figure 4.

CHASPP is used in the encoder part of the proposed machine learning model for feature extraction. With its help, the local and global features are extracted, which increases the performance of the proposed model for spine segmentation. The limitations of the ASPP are also improved with the help of CHASPP.

### 3.6. U-Net Model for Semantic Segmentation

**Figure 5:**
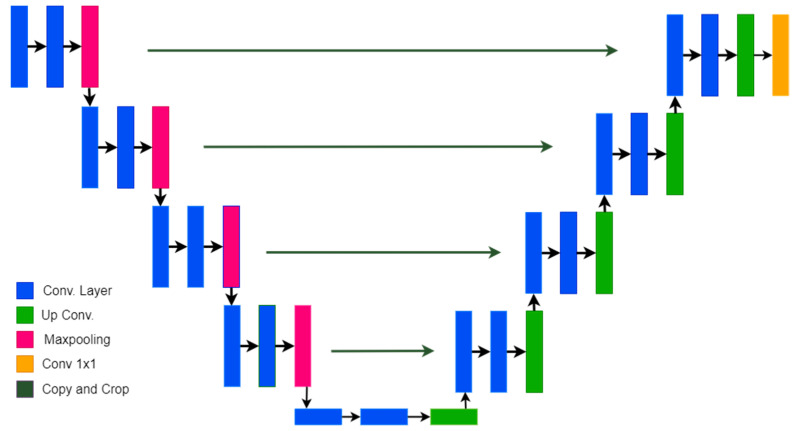
The U-Net model’s architecture for medical image segmentation.

The most popular model for the segmentation of biomedical images is U-Net [15]. It is known as U-Net because its architecture is U-shaped. It consists of two parts: an encoder and a decoder. The encoder part is used to extract features from the given dataset, and the decoder part is used to predict the segmented mask. The model uses the concept of a fully convolutional network and extracted localization as well as context features. The standard architecture of U-Net is shown in Figure 5.

The encoder block reduces the size of the image using max-pooling layers with a stride of 2. The convolutional layers are used with an increasing number of filters. In the decoder part, the number of filters starts decreasing with gradual upsampling. Skip connections that preserve the losses from previous layers and connect those layers with the layers of the decoder blocks are also used.

In the proposed approach for spine segmentation, we modified the standard U-Net architecture by adding CHASPP and residual blocks to the encoder part and adding the attention module to the decoder part. The model achieved promising results in spine segmentation and provided better segmentation.

### 3.7. Proposed Cascaded Hierarchical Atrous Spatial Pyramid Pooling Attention Residual U-Net for Spine Segmentation

**Figure 6:**
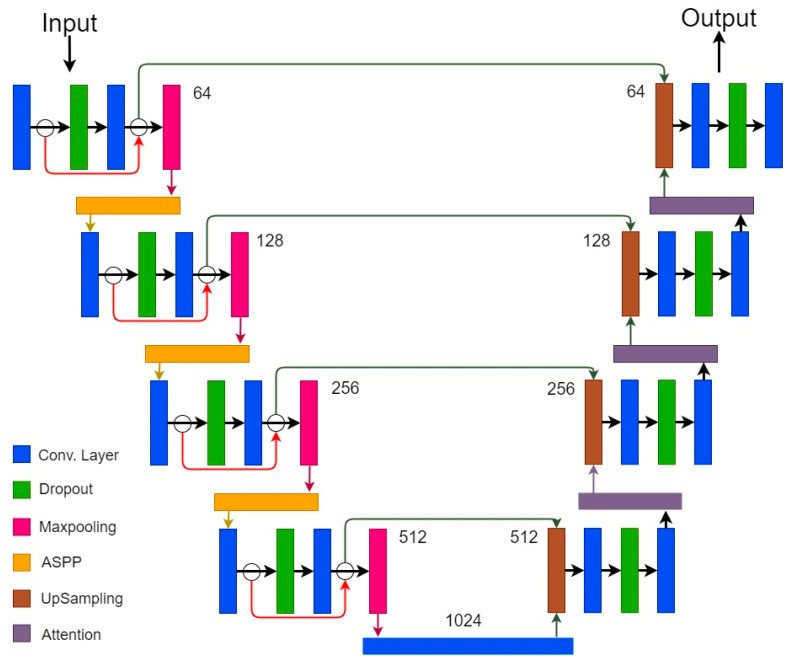
The proposed CHASPPARU-Net for automated spine segmentation.

The proposed model is a modified version of the standard U-Net architecture for the automated segmentation of the spine and was named a cascaded hierarchical atrous spatial pyramid pooling attention residual U-Net (CHASPPARU). In the standard U-Net architecture, there are convolution layers and max-pooling layers for feature extraction. The proposed model is an encoder–decoder-based architecture in which three CHASPP layers are used after each max-pooling layer in the encoder part. The complete architecture of the proposed CHASPP is shown in Figure 6. As discussed in Section 3.5, this CHASPP enhances the performance of the machine learning model by extracting local and global contextual information using multiple scale rates. Residual blocks are inserted in each encoder part that preserve the information and pass it to the decoder layers with skip connections. The decoder part of the proposed model contains attention modules that focus on the area of interest.

### 3.8. Proposed 3D Mobile Residual U-Net for Spine Vertebrae Recognition

**Figure 7:**
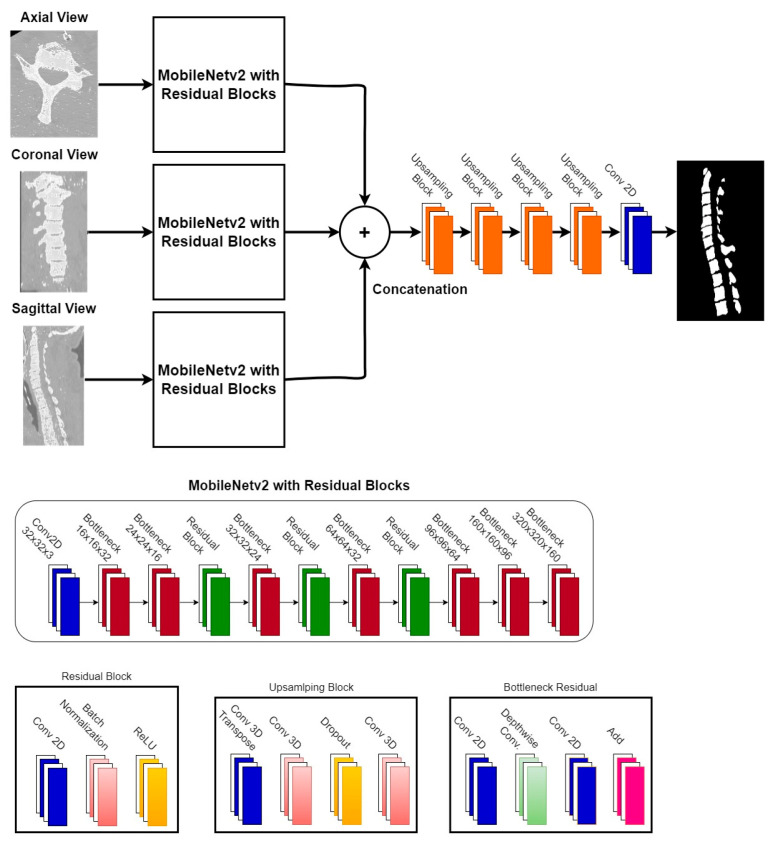
The proposed 3D MRU-Net for automated vertebrae recognition.

A 3D MRU-Net is introduced for the recognition of spine vertebrae using CT images. The complete structure of the proposed model is shown in Figure 7. The proposed model has an encoder–decoder architecture that is a combination of MobileNetV2 [36], a residual block [37], and 3D U-net [31]. MobileNetv2 is a lightweight network that can be used for low-resource devices such as mobile phones. It is difficult to process 3D images without more powerful computational resources such as GPUs. Moreover, there are a lot of trainable parameters in 3D images that further increase the computational cost. Due to these implementation challenges, a lightweight deep learning model, MobileNetv2, is used for feature extraction. The standard architecture of MobileNetv2 was modified by adding residual blocks that help in learning deep features. The new version of MobileNetv2, including residual blocks, is used as an encoder. The 3D U-net is used as a decoder for final vertebrae recognition. The input of the network is CT images of the spine, and the output is segmented spine vertebrae. The network is trained using a 2D neural network that reduces the computational cost. This approach is similar to [41], in which each orthogonal slice was trained on an individual CNN model. The three MobileNetv2 architectures are not connected to each other but are used to separately extract features from the three orthogonal slices. The output feature maps from all individual networks are similar in size and are concatenated to make a 3D feature map. This 3D feature map is given to the decoder part of the 3D-Unet for final vertebrae recognition. The evaluation of this model is conducted using evaluation metrics such as the dice score, intersection over union, precision, and recall.

### 3.9. Dataset Definition

The publicly available datasets VerSe 2020 and VerSe 2019 [42,43,44] were used in this research to evaluate the model. These challenging datasets allow researchers to adopt deep learning methods for the analysis of spines with multiple conditions, labeled vertebrae, and fields of view. The datasets can be downloaded from the OSF repository [42] and are available in the NIfTI format. The VerSe 2020 dataset consists of 300 CT images with labels. Typical anatomy such as transitional vertebrae, the sacralization of L5, and cervical ribs are included in the VerSe 2020 dataset. The VerSe 2019 data include patients with metallic implants or spinal fractures as well as combinations of isotropic and sagittal reformations. They consist of 160 CT images with centroids and segmented masks. The complete details of the VerSe 2020 and VerSe 2019 datasets are given in Table 2.

## 4. Results

### 4.1. Evaluation Metrics

The dice coefficient score, IoU, precision, and recall were used to validate the proposed models. The details of these metrics are given below:

#### 4.1.1. Dice Score

The most common and useful evaluation metric for segmentation tasks is the dice coefficient score [45]. It compares the actual mask and the predicted mask using the following formula:

*DSC* = 2(*P*_1_
*× P*_2_)/(*P*_1_ + *P*_2_) (3)

where $P_{1}$ is the predicted image and $P_{2}$ is the ground truth of the image.

#### 4.1.2. Intersection over Union (IoU)

Intersection over union [45] is commonly used to compare a predicted mask with a known mask for semantic segmentation. The formula for the calculation of *IoU* is given below:

*IoU* = *TP*/(*TP* + *FN* + *FP*) (4)

#### 4.1.3. Precision

*Precision* [45] quantifies the total number of correct positive outcomes made by the proposed model. The mathematical formula for calculating precision is given below:

*Precision* = *TP*/(*TP* + *FP*) (5)

#### 4.1.4. Recall

*Recall* [45] is calculated as the total number of true positive outcomes divided by the sum of the true positive and false negative outcomes. The mathematical formula for recall is given below:

*Recall* = *TP*/(*TP* + *FN*) (6)

### 4.2. Pre-Processing

**Figure 8:**
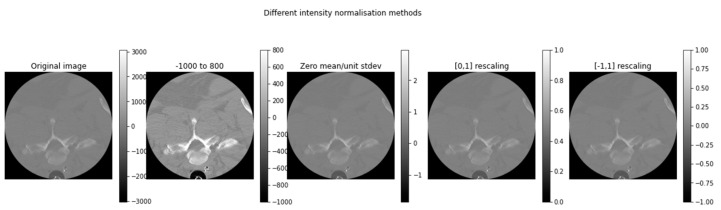
Image normalization results with rescaling between −1000 and +800, zero-mean scaling, rescaling between 0 and 1, and rescaling between −1 and 1.

The VerSe datasets were resized to 256 × 256 to reduce the computational cost of the training process. Different image normalization methods were used, as shown in Figure 8, which included zero-mean scaling, rescaling between 0 and 1, rescaling between −1 and 1, and rescaling between −1000 and +800.

The CT images from both VerSe datasets were pre-processed with smoothing, clamping, and reorienting as used by Payer et al. [26]. However, the clamping range used in this research was [−1000, 800] instead of [−1024, 8192] because the Hounsfield units (HU) of high atomic structures such as bones are in this range [43]. It is important to note that the appropriate HU threshold for bones can vary depending on the specific CT scanner and the protocol used as well as the patient’s age, sex, and other individual factors. The range of HUs in this study was selected by a radiologist with expertise in CT interpretation, who helped us to determine the most appropriate HU threshold for the dataset that was used.

The dataset was partitioned into two parts; eighty percent was used for training and twenty percent was used for testing. The proposed model was trained with 150 epochs, using 0.001 as the initial learning rate. The proposed model took 3.5 h for training and 27 s for a single prediction.

### 4.3. Experimental Results

This section shows the results of CHASPPRAU-Net and 3D MRU-Net on the VerSe 2020 and VerSe 2019 datasets for spine segmentation and vertebrae recognition. Different experiments were performed to test the performances of the proposed models. The complete details of the experiments conducted in this research are described, and the results are also compared with other methods. The results on the VerSe 2020 and VerSe 2019 datasets were obtained using the best model parameters.

The experimental results that were obtained after applying pre-processing methods are given in Table 3 and Table 4. It was concluded that the normalization range between −1000 to 800 provided the best results for both datasets when compared to other methods.

#### 4.3.1. Data Augmentation

Data augmentation is particularly important because of the limited availability of large annotated datasets. By generating new images with different variations and distortions, data augmentation can help to reduce overfitting and improve the generalizability of machine learning models. Additionally, data augmentation can help to account for differences in imaging protocols, equipment, and conditions, which can all influence the appearance of medical images. In this research, scaling, rotation, and flip rotation were used to produce augmented images. The results are given in Table 5 and Table 6.

**Figure 9:**
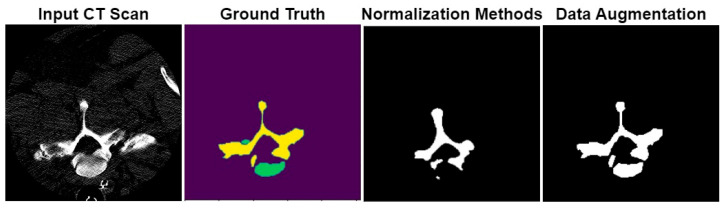
The image segmentation results of the proposed CHASPPRAU-Net model for spine segmentation.

The segmentation results of the proposed model with normalization methods and data augmentation are shown in Figure 9. From the results, it was concluded that the proposed model provides accurate segmentation results.

#### 4.3.2. Deep Feature Extraction with Residual Blocks

Residual blocks [37] are popular for extracting deep features and preserving contextual information that is lost due to convolution operations. Therefore, residual blocks were added to the encoder part of 3D MRU-Net for deep feature extraction, which improved the performance of spine vertebrae recognition. The blocks were added to MobileNetv2. The results for spine vertebrae recognition improved, as shown in Table 7.

#### 4.3.3. Dropout Regularization to Overcome Overfitting Problem

Dropout regularization is a technique used in machine learning to prevent overfitting, which occurs when a model becomes too complex and starts to fit the noise in the training data instead of the underlying patterns. During the experiments, the original dropout value of 0.3 was used, which shows a substantial increase in the prediction of the model. The results are shown in Table 8.

**Figure 10:**
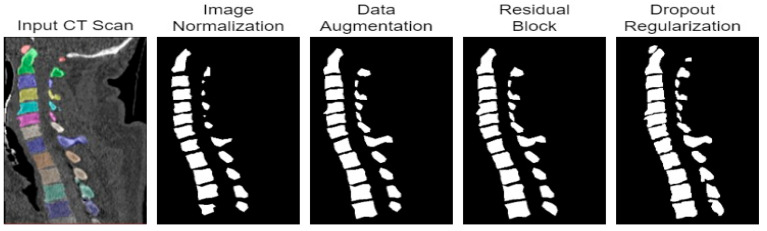
The image segmentation results of the proposed 3D MRU-Net model for spine segmentation.

The results of spine vertebrae recognition after performing all the experiments are shown in Figure 10. Qualitative comparisons of all the experiments that were conducted are shown in Table 9 and Table 10. The best results are highlighted in bold. From the results, it can be concluded that the proposed approach achieves better results for spine segmentation as well as spine vertebrae recognition, with efficient and accurate results, when compared to the state-of-the-art deep learning models.

## 5. Discussion

The results obtained using the proposed model were compared with state-of-the-art methods found in the literature using the dice score. The proposed model is efficient and robust and has low computational costs because of MobileNetv2 and the residual blocks. A comparison of the proposed model and other deep learning models is shown in Table 11, which was inspired by [32]. The model performed well with 60 test samples and achieved a dice score of 95.19%.

A 3D V-Net model was proposed by Altini et al. [44] for the automatic identification of vertebrae using k-nn, CNN, and k-means clustering. This method consists of two phases: a binary segmentation of the whole spine using a 3D network and using traditional machine learning models to find the centroids of the vertebrae. The dataset used for the testing of the model was VerSe 2020, in which 214 CT images were extracted for the training and testing of the proposed approach. This approach achieved an 89.17 percent dice score for binary segmentation and a 90.09 percent dice score for the multi-class segmentation. Kim et al. [14] proposed a web-based segmentation of the spine using a deep learning approach. The U-Net architecture was trained on a data format file for spine segmentation. There were 314 CT images in total, from which 300 images were used for training and 14 images were used for testing. The dice score achieved by this approach was 90.4 percent, and it can be used as a diagnostic tool for spine segmentation. However, the testing dataset consisted of a very small number of CT images.

Another network with a redundant class label was used for the automated segmentation of the spine [46]. This was a hybrid approach because a network was combined with a fully convolutional network to enhance the quality of model. The dataset used in this research was the spineweb dataset. The training process took 13 h to complete and achieved a dice score of 94 percent. Qadri et al. [20] introduced an automated deep learning approach using a patch-based method for learning deep features. This method selects features and measures classes. This model used only three CT images for testing and achieved a dice score of 86.1 percent. Zareie et al. [48] introduced a 3D pulse-coupled network for the segmentation of vertebrae using CT images. This model achieved a 95% score for a segmentation task validated using 17 CT images. However, more CT scan images should be used to enhance the performance of the model.

In this work, a novel deep learning approach was used for spine segmentation and vertebrae recognition. First, a CHASPPRAU-Net model was used for the segmentation of the spine. The model had an encoder–decoder architecture in which residual blocks and CHASPP modules were added to the encoder part and attention modules were added to the decoder part of the standard U-Net. After spine segmentation, a 3D MRU-Net was used for vertebrae recognition, which also had an encoder–decoder-based architecture. Three individual modified MobileNetv2 models were used on three different views of CT images (axial, coronal, and sagittal). The features from all three networks were concatenated, and a 3D feature map was given as an input to the decoder part of the 3D MRU-Net for vertebrae recognition. The VerSe 2020 and VerSe 2019 datasets were used to validate the proposed model. From the results, we can see that the proposed model achieved better results.

## 6. Conclusions

Spine segmentation and vertebrae recognition play important roles in the diagnosis process of patients. However, there is no accurate and efficient method available for the segmentation task. In this study, a novel deep learning model was proposed for the segmentation of the spine and spine vertebrae using CT images. The segmentation of the spine was performed using a CHASPPRAU-Net, while a 3D MRU-Net was used for the automated recognition of vertebrae. The performance of the proposed model was validated using different evaluation metrics. The VerSe 2020 and VerSe 2019 datasets were used for the evaluation of the model. From the results, we can see that the proposed CHASPPRAU-Net model achieved 93.72% and 94.58% dice scores for the VerSe 2020 and VerSe 2019 datasets, respectively, while the 3D MRU-Net model achieved a dice score of 95.19% for the VerSe 2019 dataset and a dice score of 93.89% for the VerSe 2020 dataset for vertebrae recognition.

## Figures and Tables

**Table 1:** The standard architecture of MobileNetv2.

Input	Operator	t	c	n	a
224^2^ × 3	Conv 2D	-	32	2	2
112^2^ × 32	Bottleneck	1	16	1	1
112^2^ × 16	Bottleneck	6	24	2	2
56^2^ × 24	Bottleneck	6	32	3	2
28^2^ × 32	Bottleneck	6	64	4	2
14^2^ × 64	Bottleneck	6	96	3	1
14^2^ × 96	Bottleneck	6	160	3	2
7^2^ × 160	Bottleneck	6	320	1	1

**Table 2:** Descriptions of VerSe 2020 and VerSe 2019 datasets.

Dataset	Spine Tract	Total Images	Modality	Annotation
VerSe 2020	Whole Spine	300	CT	Centroids and Masks
VerSe 2019	Whole Spine	160	CT	Centroids and Masks

**Table 3:** The results of the CHASPPRAU-Net model for spine segmentation using the VerSe 2020 and VerSe 2019 datasets after applying image normalization methods.

Dataset	Normalization	DSC (%)	IoU (%)	Precision (%)	Recall (%)
VerSe 2020	−1000 to 800	90.45	91.48	94.37	95.07
	Zero mean	89.91	90.34	95.16	94.32
	0 to 1	90.18	94.01	93.41	94.08
	−1 to 1	88.75	92.17	91.62	91.43
VerSe 2019	−1000 to 800	91.63	90.89	95.84	94.77
	Zero mean	88.42	89.37	93.25	92.98
	0 to 1	89.79	90.48	94.62	93.10
	−1 to 1	90.51	89.17	92.48	91.79

**Table 4:** The results of the 3D MRU-Net model for spine vertebrae recognition using the VerSe 2020 and VerSe 2019 datasets after applying image normalization methods.

Dataset	Normalization	DSC (%)	IoU (%)	Precision (%)	Recall (%)
VerSe 2020	−1000 to 800	87.96	88.39	92.76	92.46
	Zero mean	82.41	81.64	88.45	87.45
	0 to 1	83.75	84.78	89.37	88.17
	−1 to 1	82.63	83.79	86.97	85.39
VerSe 2019	−1000 to 800	86.58	87.35	90.49	91.48
	Zero mean	84.72	84.98	88.21	87.91
	0 to 1	81.15	82.47	85.79	86.48
	−1 to 1	83.49	84.09	87.89	86.12

**Table 5:** The results of the CHASPPRAU-Net model for spine segmentation using the VerSe 2020 and VerSe 2019 datasets after applying image normalization methods and data augmentation.

Dataset	Normalization	DSC (%)	IoU (%)	Precision (%)	Recall (%)
VerSe 2020	−1000 to 800	93.72	92.25	97.04	96.87
	Zero mean	92.63	93.14	96.13	94.53
	0 to 1	90.59	90.28	93.08	92.48
	−1 to 1	93.14	94.60	94.05	93.75
VerSe 2019	−1000 to 800	94.58	95.93	98.71	97.10
	Zero mean	92.49	91.68	95.62	95.78
	0 to 1	93.64	94.84	96.38	94.63
	−1 to 1	94.34	95.08	97.14	96.31

**Table 6:** The results of the 3D MRU-Net model for spine vertebrae recognition using the VerSe 2020 and VerSe 2019 datasets after applying image normalization methods and data augmentation.

Dataset	Normalization	DSC (%)	IoU (%)	Precision (%)	Recall (%)
VerSe 2020	−1000 to 800	89.59	90.78	95.27	95.88
	Zero mean	84.64	86.47	90.34	91.15
	0 to 1	85.37	84.39	92.13	90.72
	−1 to 1	84.19	85.47	89.78	88.34
VerSe 2019	−1000 to 800	90.18	91.48	96.05	95.94
	Zero mean	86.17	87.19	90.75	89.11
	0 to 1	84.65	85.94	89.19	89.78
	−1 to 1	85.48	84.97	90.12	88.95

**Table 7:** The results of the 3D MRU-Net model for spine vertebrae recognition using the VerSe 2020 and VerSe 2019 datasets after applying image normalization methods, data augmentation, and residual blocks.

Dataset	Normalization	DSC (%)	IoU (%)	Precision (%)	Recall (%)
VerSe 2020	−1000 to 800	93.46	93.66	98.71	98.47
	Zero mean	88.95	88.14	92.49	93.75
	0 to 1	89.18	90.79	95.64	95.46
	−1 to 1	88.28	88.34	91.93	92.62
VerSe 2019	−1000 to 800	94.96	95.61	98.15	97.91
	Zero mean	89.75	89.72	92.86	91.85
	0 to 1	87.48	88.63	92.45	92.14
	−1 to 1	88.96	87.98	91.43	92.48

**Table 8:** The results of the 3D MRU-Net model for spine vertebrae recognition using the VerSe 2020 and VerSe 2019 datasets after applying image normalization methods, data augmentation, residual blocks, and dropout regularization.

Dataset	Normalization	DSC (%)	IoU (%)	Precision (%)	Recall (%)
VerSe 2020	−1000 to 800	93.89	94.01	99.12	98.79
	Zero mean	89.46	90.48	92.89	94.18
	0 to 1	90.02	91.37	96.15	95.97
	−1 to 1	89.71	88.52	92.45	93.14
VerSe 2019	−1000 to 800	95.19	95.81	99.48	98.36
	Zero mean	89.92	90.72	93.62	92.34
	0 to 1	88.16	87.25	93.48	93.03
	−1 to 1	89.52	88.16	92.19	93.14

**Table 9:** A comparison of the proposed spine segmentation model with all the experiments conducted in this research on the VerSe 2020 and VerSe 2019 datasets.

Dataset	Experiments	DSC (%)	IoU (%)	Precision (%)	Recall (%)
VerSe 2020	Image Normalization	90.45	91.48	94.37	95.07
	Data Augmentation	93.72	92.25	97.04	96.87
VerSe 2019	Image Normalization	91.63	90.89	95.84	94.77
	Data Augmentation	94.58	95.93	98.71	97.10

**Table 10:** A comparison of the proposed spine vertebrae recognition model with all the experiments conducted in this research on the VerSe 2020 and VerSe 2019 datasets.

Dataset	Experiments	DSC (%)	IoU (%)	Precision (%)	Recall (%)
VerSe 2020	Image Normalization	87.96	88.39	92.76	92.46
	Data Augmentation	89.59	90.78	95.27	95.88
	Residual Blocks	93.46	93.66	98.71	98.47
	Dropout Regularization	93.89	94.01	99.12	98.79
VerSe 2019	Image Normalization	86.58	87.35	90.49	91.48
	Data Augmentation	90.18	91.48	96.05	95.94
	Residual Blocks	94.96	95.61	98.15	97.91
	Dropout Regularization	95.19	95.81	99.48	98.36

**Table 11:** Comparison of the proposed model and other deep learning models.

Reference	Methodology	Test Samples	Trainable Parameters	DSC (%)
[44]	3D V-Net	50	-	89.17
[14]	U-Net	14	1,941,105	90.40
[46]	CNN	32	-	94.28
[20]	PaDBN	3	-	86.1
[47]	3D U-Net	25	19,069,955	84.6
[48]	PCNN, APCNN, MLPNN, MLPNN1F, APCNN, MLPCNN	17	-	65.7, 95, 91.1, 77.3, 94.3, 87.8
Proposed Model	CHASPPRAU-Net, 3D MRU-Net	60	1,245,155	94.58, 95.19

## Data Availability

OSF|VerSe2020, OSF|VerSe 2019.
